# Higher-order exceptional ring semimetal with real hinge states in acoustic metamaterials

**DOI:** 10.1093/nsr/nwag221

**Published:** 2026-04-10

**Authors:** Yejian Hu, Zhenhang Pu, Xiangru Chen, Yuxiang Xi, Jiuyang Lu, Weiyin Deng, Manzhu Ke, Zhengyou Liu

**Affiliations:** Key Laboratory of Artificial Micro- and Nanostructures of Ministry of Education and School of Physics and Technology, Wuhan University, Wuhan 430072, China; Key Laboratory of Artificial Micro- and Nanostructures of Ministry of Education and School of Physics and Technology, Wuhan University, Wuhan 430072, China; Key Laboratory of Artificial Micro- and Nanostructures of Ministry of Education and School of Physics and Technology, Wuhan University, Wuhan 430072, China; Key Laboratory of Artificial Micro- and Nanostructures of Ministry of Education and School of Physics and Technology, Wuhan University, Wuhan 430072, China; Key Laboratory of Artificial Micro- and Nanostructures of Ministry of Education and School of Physics and Technology, Wuhan University, Wuhan 430072, China; Key Laboratory of Artificial Micro- and Nanostructures of Ministry of Education and School of Physics and Technology, Wuhan University, Wuhan 430072, China; Key Laboratory of Artificial Micro- and Nanostructures of Ministry of Education and School of Physics and Technology, Wuhan University, Wuhan 430072, China; Key Laboratory of Artificial Micro- and Nanostructures of Ministry of Education and School of Physics and Technology, Wuhan University, Wuhan 430072, China; Institute for Advanced Studies, Wuhan University, Wuhan 430072, China

**Keywords:** exceptional ring, higher-order topology, non-Hermitian systems, acoustic metamaterials

## Abstract

Non-Hermitian topological phase, with the novel concepts such as exceptional points and the skin effect, has opened up a new paradigm beyond Hermitian topological physics. Exceptional ring semimetal, featured by a stable ring of exceptional points in three dimensions, exhibits first-order topological properties, including topological surface states and a surface-dependent skin effect. Nevertheless, despite extensive research on Hermitian higher-order insulators and semimetals, higher-order exceptional ring semimetal is just emerging. Here, we report the first realization of a higher-order Weyl exceptional ring semimetal in a three-dimensional lossy acoustic metamaterial. The non-Hermitian higher-order topology is reflected in the topological hinge states and a hinge-dependent skin effect. Counterintuitively, the topological hinge states maintain purely real energy even under a high loss level, ensuring robust hinge-state propagation. Our findings evidence the non-Hermitian higher-order bulk-boundary correspondence of exceptional ring semimetal, and may pave the way for non-Hermitian functional acoustic devices.

## Teaser

A higher order Weyl exceptional ring semimetal wa s realized in a three-dimensional lossy acoustic metamaterial, featuring topological real hinge states and a hinge dependent skin effect.

## INTRODUCTION

Topological physics within the Hermitian paradigm has drawn substantial attention [1]. Topological semimetals are featured by nontrivial bulk band crossings [2]. For instance, the renowned Weyl semimetals exhibit doubly degenerate band crossings in three-dimensional (3D) momentum space, referred to as Weyl points (WPs). The WPs behave as monopoles of Berry flux and possess topological charges characterized by Chern numbers. In consequence, Weyl semimetals host ensured Fermi arc surface states that connect the projections of oppositely charged WPs [2,3]. Over the past years, the growing interest in higher-order topology arose first in insulating phases [4,5] and then in 3D semimetal phases [6–9], including the Weyl semimetals. Unlike conventional or first-order topological semimetals, higher-order Weyl semimetals are identified by topological hinge states (THSs) on their 1D boundaries [9,10]. Benefiting from the macroscopic scale and the flexibility in design of classical artificial metamaterials [11–13], higher-order Weyl semimetals have been experimentally confirmed in acoustic [14], optical [15], and circuit metamaterials [16], revealing the hierarchical nature of Weyl topology.

Recently, it has been discovered that the scope of topological physics can be dramatically expanded in non-Hermitian systems. Non-Hermitian topological phases exhibit a wealth of unique phenomena [17–27], including exceptional degeneracies [17–19], spectral braiding [19–21], and skin effects [22–25]. Particularly noteworthy are non-Hermitian topological semimetals, which host exceptional degenerate points [28,29] or lines [30–32] with coalesced eigenstates [33–35], attracting significant research attention in the past years [36–39]. Among them, Weyl exceptional ring (WER) semimetals are characterized by exceptional doubly degenerate rings, termed WERs, which can be evolved from the WPs under non-Hermitian perturbations [40–43]. WER inherits the topological charge of WP behaving as a line source or sink of Berry flux, and acquires a new topological charge of spectral winding number [44–48] without Hermitian counterpart. Intriguingly, WERs are discovered to support THSs as well, bringing to light the first type of non-Hermitian higher-order topological semimetal in theory [49]. Compared with extensive research on Hermitian higher-order insulators and semimetals, investigation into non-Hermitian higher-order topological semimetal is critically lacking, especially in experiments [49,50].

Here, we report the first realization of a higher-order WER semimetal in a 3D non-Hermitian acoustic metamaterial (AM), which is developed from a stacked breathing Kagome lattice with loss. The AM hosts WERs with dual topological charges in terms of quantized Chern number and spectral winding number, as the first-order topologies, giving rise to Fermi arc surface states and surface-dependent skin effect, respectively. More importantly, the second-order topologies are revealed by the THSs protected by the bulk polarization, and the hinge-dependent skin effect stemming from the interplay between the Fermi arc surface states and non-Hermitian skin effect. Unlike the almost unobservable trivial hinge states, the WER-induced THSs maintain counterintuitive purely real energy, which ensures robust hinge propagation for acoustic waves against introduced loss. The theoretical, simulated, and experimental results exhibit a high degree of concordance.

## RESULTS AND DISCUSSION

**Figure 1.**
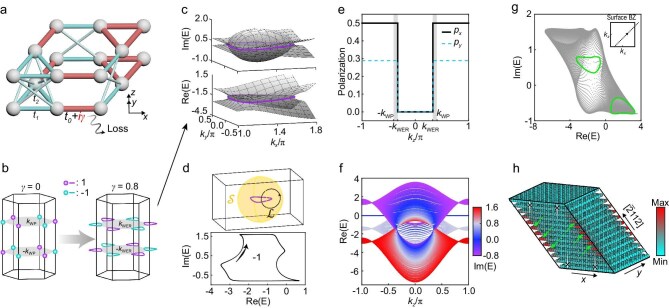
Second-order WER semimetal for a 3D stacked Kagome lattice. (a) Schematic of the 3D lattice in a rhombic prism geometry, with three sublattices in a unit cell. Loss is added in the intercell hopping ${t}_0 + i\gamma $, while ${t}_1$ and ${t}_2$ are Hermitian. (b) Distributions of the WPs (left panel) and WERs (right panel) in the BZ. The topological charges of Chern numbers are indicated by the purple ($+ 1$) and cyan ($- 1$) colors. (c) Real and imaginary parts of the local dispersion around one WER in (b), as marked by the black arrow. (d) Top panel: a WER enclosed by a surface $\mathcal{S}$ (yellow sphere) and encircled by a path $\mathcal{L}$ (black circle). Bottom panel: complex energy spectrum for the first two bands along $\mathcal{L}$. (e) Bulk polarization $( {{p}_x,{p}_y} )$ of the lowest band as a function of ${k}_z$. (f) Real part of the hinge dispersion along the ${k}_z$ direction, with the imaginary part given by the colormap. (g) Complex energy spectrum for the XZ surface states, along the route ${k}_z = {k}_x - \pi /3$ in the surface BZ (dashed line in the inset). The gray and green dots represent the bulk and surface states, respectively. (h) Hinge-dependent skin effect, where the XZ surface states selectively accumulate on $[ {\bar{2}112} ]$-directional hinges. The parameters are chosen as ${t}_0 = - 1.385$, ${t}_1 = - 1$, ${t}_2 = - 0.6$ and $\gamma = 0.8$.

We start from a three-band lattice model in Fig. 1(a), which is constructed by stacking the breathing Kagome lattice along the *z* direction via cross-linked hoppings. The tight-binding Hamiltonian in momentum space reads


(1) \begin{eqnarray*}
H = \left( {\begin{array}{@{}c@{}c@{\quad}c@{}} 0&\qquad {{J}_1 + {J}_0{e}^{ - i\left( {\frac{{{k}_x}}{2} + \frac{{\sqrt 3 {k}_y}}{2}} \right)}}&\quad {{J}_1 + {J}_0{e}^{ - i{k}_x}}\\ {{J}_1 + {J}_0{e}^{i\left( {\frac{{{k}_x}}{2} + \frac{{\sqrt 3 {k}_y}}{2}} \right)}}&\quad 0&\quad {{J}_1 + {J}_0{e}^{ - i\left( {\frac{{{k}_x}}{2} - \frac{{\sqrt 3 {k}_y}}{2}} \right)}}\\
{{J}_1 + {J}_0{e}^{i{k}_x}}&\quad {{J}_1 + {J}_0{e}^{i\left( {\frac{{{k}_x}}{2} - \frac{{\sqrt 3 {k}_y}}{2}} \right)}}&\quad 0 \end{array}} \right),
\end{eqnarray*}

with ${J}_1 = {t}_1 + 2{t}_2\cos {k}_z$, ${J}_0 = {t}_0 + i\gamma $, and ${k}_i$  $( {i = x,y,z} )$ are the dimensionless wave vectors. Loss is added in the intercell hopping ${t}_0 + i\gamma $ and can bring the non-Hermiticity, and other hoppings are Hermitian. For $\gamma = 0$, the system is Hermitian with real energies and hosts four WPs between the first two bands, as shown in the left panel of Fig. 1(b). These WPs reside at the corners of the ${k}_z = \pm {k}_{{\mathrm{WP}}}$ planes (${k}_{{\mathrm{WP}}} = {\mathrm{acos}}[ {( {{t}_0 - {t}_1} )/2{t}_2} ]$) in the first Brillouin zone (BZ), carrying topological charges of Chern numbers $\pm 1$. When increasing $\gamma $, non-Hermiticity is introduced, and each WP evolves into a WER located at the ${k}_z = \pm {k}_{{\mathrm{WER}}}$ planes (${k}_{{\mathrm{WER}}} = {\mathrm{acos}}[ {( { - \sqrt {t_0^2 + {\gamma }^2} - {t}_1} )/2{t}_2} ]$), as shown in the right panel of Fig. 1(b). The local dispersion around a WER is visible in Fig. 1(c), and the simultaneous degeneracy of the real and imaginary parts of bands only occurs at the WER. When loss is applied to other couplings, the WPs also evolve into WERs, connecting the real- and imaginary-part degeneracy surfaces, as detailed in Section S1.

Now, we characterize the topological properties of the WERs. Originating from the interplay between the WPs and non-Hermiticity, the WERs carry dual topological charges described by Chern numbers and spectral winding numbers. The Chern number *C* is defined as the integration of the Berry curvature over a closed surface $\mathcal{S}$ (yellow sphere in the top panel of Fig. 1(d)):


(2) \begin{eqnarray*}
C = \frac{1}{{2\pi i}}\mathop \oint \nolimits_\mathcal{S} {\nabla }_{\boldsymbol{k}} \times \left\langle {\tilde{u}\left( {\boldsymbol{k}} \right){\mathrm{|}}{\nabla }_{\boldsymbol{k}}{\mathrm{|}}u\left( {\boldsymbol{k}} \right)} \right\rangle \cdot {\rm d}{{\bf S}},
\end{eqnarray*}

where ${|u( {\boldsymbol{k}} )} \rangle $ (${|\tilde{u}( {\boldsymbol{k}} )} \rangle $) is the left (right) eigenvector for the first band. Naturally, the WERs inherit the nonzero $C = \pm 1$ from the WPs, as marked by the purple and cyan colors in Fig. 1(b). Besides, the winding number *v* labels the spectral topology for the first two bands along a closed path $\mathcal{L}$ encircling the WER (black circle in top panel of Fig. 1(d)), defined as


(3) \begin{eqnarray*}
v = \frac{1}{{2\pi }}\mathop \sum \limits_{i,j = 1}^2 \mathop \oint \nolimits_\mathcal{L} {\rm d}{\boldsymbol{k}} \cdot {\nabla }_{\boldsymbol{k}}\arg \left[ {{E}_i\left( {\boldsymbol{k}} \right) - {E}_j\left( {\boldsymbol{k}} \right)} \right],\\
\end{eqnarray*}

where ${E}_{i( j )}$ represents the energy of the *i*- (*j*-)th band. For example, $v = - 1$ indicates that the first two bands wind clockwise in the complex plane and form a point gap, as exemplified in the bottom panel of Fig. 1(d). Numerical calculations show that the winding numbers of the WERs at the ${k}_z = \pm {k}_{{\mathrm{WER}}}$ planes take values of $v = \mp 1$, as further verified by detailed calculations in Section S3. Owing to the dual topological charges, the Fermi arc surface states and surface-dependent skin effect are ensured, as detailed in Sections S2 and S4.

To demonstrate the second-order topology, we regard our 3D system as a set of ${k}_z$-dependent 2D subsystems and investigate the second-order topological index, that is, the bulk polarization, defined based on the 2D BZ:


(4) \begin{eqnarray*}
{p}_{x,y}\left( {{k}_z} \right) = \frac{1}{S}\,\mathop \int\!\!\!\!\!\!\int \limits_{2{\mathrm{D\ BZ}}} - i\left\langle {\tilde{u}\left( {\boldsymbol{k}} \right){\mathrm{|}}{\partial }_{{k}_{x,y}}{\mathrm{|}}u\left( {\boldsymbol{k}} \right)} \right\rangle {\rm d}^2k,\\
\end{eqnarray*}

where *S* is the area of the 2D BZ. Figure 1(e) shows the evolution of the bulk polarization for the first band as a function of ${k}_z$, a nontrivial $( {{p}_x,{p}_y} ) = ( {1/2,1/2\sqrt 3 } )$ emerges for $| {{k}_z} | > {k}_{{\mathrm{WER}}}$ within the hinge BZ and indicates the presence of THSs in this region. To verify the THSs, we demonstrate the hinge dispersion in Fig. 1(f), with its imaginary part given by the colormap. As expected, a branch of zero-energy THS dispersion emerges, which is localized at the left lower hinge of the geometry in Fig. 1(a) owing to the bulk-hinge correspondence [51]. Notably, the bulk polarization remains unaffected by increasing loss and its phase transition points in ${k}_z$ always coincide with the positions of the WPs/WERs/ERs, as detailed in Section S5.

As a second-order skin effect, the hinge-dependent skin effect is further discussed in our model. Without loss of generality, we focus on the XZ surfaces and calculate the energy spectrum of a ribbon structure with periodic boundary conditions in the *x* and *z* directions. As shown in Fig. 1(g), the surface states (green dots) along a route ${k}_z = {k}_x - \pi /3$ in the surface BZ form two point gaps, with the left one corresponding to Fermi arc surface states ensured by the WERs. That means the XZ surface states can accumulate along the ${k}_z = {k}_x$ direction under fully open boundary conditions and localize at $[ {\bar{2}112} ]$-directional hinges as skin modes, as shown in Fig. 1(h). The colormap represents the distribution of the XZ surface states defined as ${W}_s( j ) = \frac{1}{{{N}_s}}\mathop \sum \nolimits_{{n}_s} {| {{\psi }_{{n}_s}( j )} |}^2$, where ${\psi }_{{n}_s}( j )$ is the ${n}_s$th right eigenstate at site *j* and ${N}_s$ denotes the total number of surface states. However, due to the mirror symmetries along the *x* and *z* directions, such skin modes are selectively forbidden on horizontal and vertical hinges. In other words, the second-order skin effect here is hinge-dependent, as depicted by the arrows in Fig. 1(h), which is significantly different from 2D scenarios (see details in Section S4).

**Figure 2.**
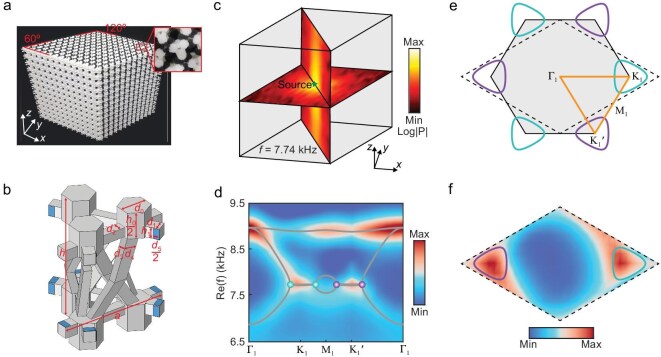
Acoustic realization of the second-order WER semimetal and observation of the WERs. (a) Photograph of the non-Hermitian AM sample. The inset gives the enlarged top view. (b) Schematic of the AM unit cell. The blue areas represent designed rectangular holes, which are sealed with sponges and introduce loss. (c) Experimental results for the WER observation. The colormaps exemplify two slices of the measured acoustic field inside the sample at $7.74\ {\mathrm{kHz}}$. (d) Measured (colormap) and simulated (gray lines) bulk dispersion along the orange high-symmetry lines depicted in (e). The colored spheres represent exceptional points in the WERs. (e) Simulated configuration of the acoustic WERs on the ${k}_z = - {k}_{{\mathrm{WER}}}$ plane. (f) Measured isofrequency contour (colormap) at the WER frequency, corresponding to the dashed rhombus in (e).

Then, we implement the aforementioned model in a 3D non-Hermitian AM. Figure 2(a) gives the photographs of the 3D-printed AM sample, which is a rhombic prism containing $13 \times 13 \times 13$ unit cells with the in-plane and out-of-plane lattice constants $a = 40\ {\mathrm{mm}}$ and $h = 45.87\ {\mathrm{mm}}$. In each unit cell, as illustrated in Fig. 2(b), there are three hexagonal prism cavities of height ${h}_0 = 20\ {\mathrm{mm}}$ and width ${d}_0 = 10\ {\mathrm{mm}}$, which are filled with air and correspond to the sites in the lattice model. The connecting tubes between the cavities mimic the hoppings. Specifically, the staggered intralayer hoppings are introduced by cuboid tubes with ${d}_1 = 4.2\ {\mathrm{mm}}$ and ${d}_2 = 3.5\ {\mathrm{mm}}$, while the interlayer hoppings are enabled by cross-linked tubes with ${d}_3 = 5/\sqrt 3 \ {\mathrm{mm}}$ and ${d}_4 = 5\ {\mathrm{mm}}$. To add loss in our sample, sponge-filled rectangular holes (blue areas in Fig. 2(b), ${d}_5 = 4.4\ {\mathrm{mm}}$ and ${h}_1 = 3.8\ {\mathrm{mm}}$) are delicately designed on the intercell tube walls (see Section S6 for details). In simulations, impedance boundaries of $1500\ {\mathrm{Pa\ s}}/{\mathrm{m}}$ are applied to these blue areas [52], and hard boundaries to the gray regions considering the huge acoustic impedance mismatch between the 3D-printed plastic material and air. Enlarged configuration of the sponges (black) is also provided in the inset of Fig. 2(a). It is worth noting that, to distinguish and detect the bulk, surface, and hinge dispersions in experiments, we construct the sample in the geometry in Fig. 1(a) where skin modes are forbidden. Simulated results for the spectral topology of acoustic WERs are given in Section S7. The surface-dependent and hinge-dependent skin effects for acoustic waves are presented in Section S8.

We demonstrate the acoustic WER by measuring the bulk band structure of the AM sample. Figure 2(c) illustrates the experimental measurement. A broadband point source (green star) is placed at the center of the sample to excite the bulk states, and the acoustic signals are recorded by a microphone inserted into the cavities. Two slices of the measured acoustic field are exemplified by the colormaps in Fig. 2(c), and one can see that the wave propagation in the *x*–*y* plane is weaker than that along the *z* direction, which is due to the loss added in the intralayer hoppings. By Fourier transforming the 3D acoustic field, we obtain the band structure of the AM sample. Focusing on the ${k}_z = - {k}_{{\mathrm{WER}}}$ plane, we display the bulk dispersion in Fig. 2(d), where two WERs are clearly observed at the frequency $f = 7.74\ {\mathrm{kHz}}$. The gray lines and colormap represent the simulated and measured results, respectively. The corresponding high symmetry route is depicted by the orange line in Fig. 2(e), which passes through the WERs at four exceptional points round ${K}_1$ and $K_1^{\prime}$. These crossing points are highlighted by purple and cyan spheres in Fig. 2(d). For the region inside the WERs, the first two bands are relatively flat but not degenerate, consistent with the theoretical prediction. In Fig. 2(f), we further display the isofrequency contour at the WER frequency, where the triangle-like shape of the WERs and the relatively flat bands are commendably verified.

**Figure 3.**
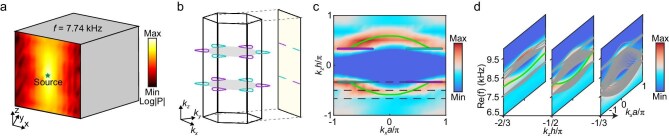
Observation of the Fermi arc surface states. (a) Experimental result for the surface states observation. The colormap represents the measured surface field at $7.74\ {\mathrm{kHz}}$. (b) Projections of the WERs in the surface BZ of the XZ surface. (c) Measured (colormap) and simulated (green lines) isofrequency contour of the surface waves at the WER frequency, where the surface states connect the projections of WERs with opposite Chern numbers. (d) Measured surface dispersions (colormap) in terms of discretized ${k}_z$, corresponding to the black dashed lines in (c). Simulated bulk and surface states are denoted by gray and green lines, respectively.

As a significant signature of WER semimetals, the topologically ensured Fermi arc surface states are validated in our AM. Figure 3(a) gives the experimental measurements. The source (green star) is centered at the front XZ surface, and the Fermi arc surface states are experimentally accessible from the Fourier transformation of the surface acoustic field (colormap). The corresponding surface BZ is shown in Fig. 3(b), where the projected WERs are restricted to the purple and cyan lines. Owing to the first-order topological nature of the WERs, two open arcs of topological surface states emerge in the isofrequency contour in Fig. 3(c), connecting the projected WERs with opposite Chern numbers. The dashed lines label specific ${k}_z = - 2/3$, $- 1/2$, $- 1/3\ \pi /h$, with the corresponding surface dispersions presented in Fig. 3(d). The gray and green lines denote simulated bulk and surface states, respectively. Note that, due to the zero ${k}_z$-dependent Chern number, the surface states do not touch the bulk states for some ${k}_z$, for example, in the left panel of Fig. 3(d), but are gapless as a whole.

**Figure 4.**
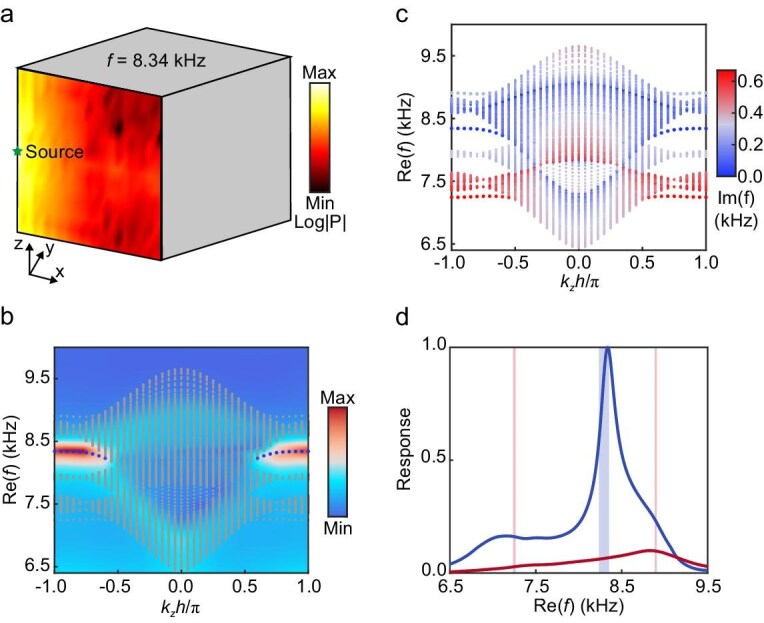
Observation of the hinge states. (a) Experimental result for the hinge states observation. The colormap represents the measured hinge field at $8.34\ {\mathrm{kHz}}$. (b) Measured hinge dispersion (colormap) for the left hinge. Simulated result is denoted by gray dots and the THSs are highlighted in blue. (c) Real part of the simulated hinge dispersion of the AM sample, where the imaginary part is given by the colormap. The THSs around $8.34\ {\mathrm{kHz}}$ are localized at the left hinge, and the trivial hinge states around ${7.24}$ and $8.90\ {\mathrm{kHz}}$ are localized at the right hinges. (d) Measured response spectra for the left (blue line) and right (red line) hinges of the AM sample. The blue and red regions label the frequency ranges of the THSs and trivial hinge states, respectively.

To validate the second-order topology in our AM, experimental measurement for the THSs is shown in Fig. 4(a). One can see from the colormap that, with respect to a source (green star) centered at the left hinge, the acoustic wave is localized at the hinge and propagates along the *z* direction. Obtained by Fourier transforming the 1D acoustic field at the hinge, the hinge dispersion of the AM sample is given by the colormap in Fig. 4(b). Because of the nontrivial bulk polarization, a branch of THS dispersion emerges at around $8.34\ {\mathrm{kHz}}$ in the region of $| {{k}_z} | > {k}_{{\mathrm{WER}}}$ as expected, identifying our AM as a second-order WER semimetal. The dots display the simulated result for comparison, agreeing with the experimental data well. Owing to their protection by a nonzero bulk polarization, the THSs exhibit robustness against random disorders, as discussed in Section S9.

Besides the THSs, there are two branches of trivial hinge states at around $7.24$ and $8.90\ {\mathrm{kHz}}$, which are localized at the right hinge of the AM sample. To be more explicit, we display the simulated hinge dispersion in Fig. 4(c) with the imaginary parts labeled by colormap. One can see that the trivial hinge states have larger imaginary parts of frequencies, implying that the hinge wave propagation is influenced by the system loss. On the contrary, the THSs have negligible imaginary parts of frequencies, revealing their robustness against non-Hermitian perturbations. This fact can be confirmed by the response spectra for acoustic waves propagating along different hinges of our sample, as shown in Fig. 4(d), which are measured by exciting and probing two adjacent cavities on the corresponding hinges. The response curve for the left hinge (blue line) reaches a strong peak in the blue region, representing the THSs, while the curve for the right hinge (red line) has a much weaker intensity. Experimental data for trivial hinge states can be found in Section S10.

## CONCLUSION

In summary, we have realized a second-order WER semimetal in a 3D AM, where the non-Hermiticity is introduced by designed loss. The acoustic WERs carry dual topological charges that result in Fermi arc surface states and surface-dependent skin effect, and the second-order topologies are reflected by robust 1D THSs and hinge-dependent skin effect. Notably, given the unavoidable intrinsic loss in natural and engineered systems, the WER-induced real hinge states hold significant potential for applications in topological waveguides and sensors. Our work broadens the insight into the interplay between topological semimetal and non-Hermiticity, and may pave the way for the development of non-Hermitian acoustic devices.

## METHODS

Detailed methods can be found in the online Supplementary file.

## Supplementary Material

nwag221_Supplemental_File
