# Supplementary material for: Higher-order exceptional ring semimetal with real hinge states in acoustic metamaterials
Source: Natl Sci Rev. 2026 Apr 10;13(11):nwag221. doi: 10.1093/nsr/nwag221 (PMC13291824; doi:10.1093/nsr/nwag221)
Supplement: nwag221_Supplemental_File [file nwag221_supplemental_file.pdf]

Supplementary Materials for

Higher-order exceptional ring semimetal with real hinge states in acoustic metamaterials

Yejian Hu[1†], Zhenhang Pu[1†], Xiangru Chen[1], Yuxiang Xi[1], Jiuyang Lu[1], Weiyin Deng[1*], Manzhu Ke[1*], and Zhengyou Liu[1,2*]

[1]Key Laboratory of Artificial Micro- and Nanostructures of Ministry of Education and School of Physics and Technology, Wuhan University, Wuhan 430072, China

[2]Institute for Advanced Studies, Wuhan University, Wuhan 430072, China

†These authors contributed equally to this work.

*Corresponding author.

Emails: dengwy@whu.edu.cn; mzke@whu.edu.cn; zyliu@whu.edu.cn

S1. Spectral degeneracy

S2. Fermi arc surface states

S3. Spectral topology of ERs with different loss strengths

S4. Non-Hermitian skin effects

S5. Bulk polarization with different loss strengths

S6. Designed loss in AM sample

S7. Spectral winding number of the acoustic WERs

S8. Non-Hermitian skin effects for acoustic waves

S9. Robustness of the topological hinge states against perturbations

S10. Observation of the trivial hinge states

S11. Methods

S1. Spectral degeneracy

In this section, we show the spectral degenerate features of the Weyl exceptional ring (WER) semimetal. First, we analyze the scenario where loss is applied to the coupling term t_0, which corresponds to the specific case discussed in this work. According to the tight-binding Hamiltonian in Eq. (1) of main text, the eigenvalues can be obtained as

$$\begin{cases} E_{1,2} = \frac{1}{2}\left[J_0 + J_1 \mp \sqrt{\varepsilon}\right] \\ E_3 = -(J_0 + J_1) \end{cases}, \tag{S1}$$

where $\varepsilon = 9J_0^2 - 6J_0J_1 + 9J_1^2 + 8J_0J_1(\cos k_x + 2\cos\frac{k_x}{2}\cos\frac{\sqrt{3}k_y}{2})$, $J_0 = t_0 + i\gamma$ and $J_1 = t_1 + 2t_2\cos k_z$, with t_0, t_1, and t_2 being negative real numbers and γ being a positive real number. Using Euler's formula, the term $\sqrt{\varepsilon}$ in $E_{1,2}$ can be written as

$$\sqrt{\varepsilon} = \sqrt{F_1 + iF_2} = \sqrt{Re^{i\theta}} = \sqrt{R}(\cos\frac{\theta}{2} + i\sin\frac{\theta}{2}), \tag{S2}$$

where the parameters F_1 and F_2 are

$$\begin{cases} F_1 = 9t_0^2 - 6t_0J_1 + 9J_1^2 - 9\gamma^2 + 8t_0J_1\left(\cos k_x + 2\cos\frac{k_x}{2}\cos\frac{\sqrt{3}k_y}{2}\right) = R\cos\theta \\ F_2 = -6\gamma J_1 + 18\gamma t_0 + 8\gamma J_1\left(\cos k_x + 2\cos\frac{k_x}{2}\cos\frac{\sqrt{3}k_y}{2}\right) = R\sin\theta \end{cases}, \tag{S3}$$

with R being a positive real number and $\theta \in (0,2\pi]$. From Eq. S2, the real-part-degeneracies between the first two bands $E_{1,2}$ require $\cos\frac{\theta}{2} = 0$, i.e., $\theta = \pi$, which means $F_1 = -R \leq 0$ and $F_2 = 0$, i.e.,

$$\begin{cases} 9t_0^2 - 6t_0J_1 + 9J_1^2 - 9\gamma^2 + 8t_0J_1\left(\cos k_x + 2\cos\frac{k_x}{2}\cos\frac{\sqrt{3}k_y}{2}\right) \leq 0 \\ -6\gamma J_1 + 18\gamma t_0 + 8\gamma J_1\left(\cos k_x + 2\cos\frac{k_x}{2}\cos\frac{\sqrt{3}k_y}{2}\right) = 0 \end{cases}. \tag{S4}$$

The second line of Eq. S4 can be simplified as

$$\cos k_x + 2\cos\frac{k_x}{2}\cos\frac{\sqrt{3}k_y}{2} = \frac{3J_1 - 9t_0}{4J_1}. \tag{S5}$$

Plug Eq. S5 into the first line of Eq. S4, we can get

$$|t_1 + 2t_2\cos k_z| \leq \sqrt{t_0^2 + \gamma^2}. \tag{S6}$$

To obtain the detailed range of k_z, we need to conduct a discussion by cases. In the first case with $\cos k_z \geq -\frac{t_1}{2t_2}$, $t_1 + 2t_2\cos k_z < 0$. Therefore, Eq. S6 can be

simplified as

$$-t_1 - 2t_2 \cos k_z \le \sqrt{t_0^2 + \gamma^2} \Rightarrow \cos k_z \le -\frac{\sqrt{t_0^2+\gamma^2}+t_1}{2t_2}.$$

In the second case with $\cos k_z < -\frac{t_1}{2t_2}$, $t_1 + 2t_2 \cos k_z > 0$. Eq. S6 can be simplified as

$$t_1 + 2t_2 \cos k_z \le \sqrt{t_0^2 + \gamma^2} \Rightarrow \cos k_z \ge \frac{\sqrt{t_0^2+\gamma^2}-t_1}{2t_2}.$$

Since k_x, k_y are real wave vectors, there is an additional restrictive condition in Eq. S5

$$-1.5 \le \cos k_x + 2\cos\frac{k_x}{2}\cos\frac{\sqrt{3}k_y}{2} = \frac{3J_1 - 9t_0}{4J_1} \le 3$$

$$\Rightarrow -1 \le \frac{t_1+2t_2\cos k_z - 3t_0}{2t_1+4t_2\cos k_z} \le 2. \qquad \text{(S7)}$$

In the first case with $\cos k_z \ge -\frac{t_1}{2t_2}$. Eq. S7 can be simplified as

$$\cos k_z \ge \frac{t_0-t_1}{2t_2}.$$

In the second case with $\cos k_z < -\frac{t_1}{2t_2}$. Eq. S7 can be simplified as

$$\cos k_z \le \frac{-t_0-t_1}{2t_2}.$$

Combining Eq. S6 and Eq. S7, the distribution of real-part degeneracy is divided into two parts. The first part of the real-part-degeneracies is described by

$$\begin{cases} \frac{t_0-t_1}{2t_2} \le \cos k_z \le -\frac{\sqrt{t_0^2+\gamma^2}+t_1}{2t_2} \\ \cos k_x + 2\cos\frac{k_x}{2}\cos\frac{\sqrt{3}k_y}{2} = \frac{3J_1-9t_0}{4J_1} \end{cases}. \qquad \text{(S8)}$$

The second part of the real-part-degeneracies is described by

$$\begin{cases} \frac{\sqrt{t_0^2+\gamma^2}-t_1}{2t_2} \le \cos k_z \le \frac{-t_0-t_1}{2t_2} \\ \cos k_x + 2\cos\frac{k_x}{2}\cos\frac{\sqrt{3}k_y}{2} = \frac{3J_1-9t_0}{4J_1} \end{cases}. \qquad \text{(S9)}$$

The parameters selected in this work satisfy $\frac{-t_0-t_1}{2t_2} < -1$; thus, the real-part degeneracy is only described by Eq. S8, as shown by the red surfaces in Fig. S1(a). The first line of Eq. S8 decides the range of the real-part-degenerate surfaces along the k_z

direction in the first Brillouin zone (BZ), and the second line describes the ring-like curves with fixed k_z.

The imaginary-part degeneracies require $\sin\frac{\theta}{2} = 0$, i.e., $\theta = 0$; similarly, its degenerate distribution is divided into two parts.

$$\begin{cases} \cos k_z \geq -\frac{\sqrt{t_0^2+\gamma^2}+t_1}{2t_2} \\ \cos k_x + 2\cos\frac{k_x}{2}\cos\frac{\sqrt{3}k_y}{2} = \frac{3J_1-9t_0}{4J_1} \end{cases}. \tag{S10}$$

The second part of the imaginary-part-degeneracies is described by

$$\begin{cases} \cos k_z \leq \frac{\sqrt{t_0^2+\gamma^2}-t_1}{2t_2} \\ \cos k_x + 2\cos\frac{k_x}{2}\cos\frac{\sqrt{3}k_y}{2} = \frac{3J_1-9t_0}{4J_1} \end{cases}. \tag{S11}$$

The parameters selected in this work satisfy $\frac{\sqrt{t_0^2+\gamma^2}-t_1}{2t_2} < -1$; thus, the imaginary-part degeneracy is only described by Eq. S10, as shown by the blue surfaces in Fig. S1(a). Notably, the boundary curves connecting the real- and imaginary-part degeneracy surfaces correspond to WERs, which are described by

$$\cos k_x + 2\cos\frac{k_x}{2}\cos\frac{\sqrt{3}k_y}{2} = C_0, \tag{S12}$$

where $C_0 = \frac{3(t_1+2t_2\cos k_{z0})-9t_0}{4(t_1+2t_2\cos k_{z0})}$ with $\cos k_{z0} = -\frac{\sqrt{t_0^2+\gamma^2}+t_1}{2t_2}$. In other words, the WERs are located at $k_z = \pm\operatorname{acos}\frac{-\sqrt{t_0^2+\gamma^2}-t_1}{2t_2}$ planes. Note that, when $\gamma = 0$, the Weyl points (WPs) locate at the $k_z = \pm\operatorname{acos}\frac{t_0-t_1}{2t_2}$ plane, which is on the real-part-degenerate surfaces for arbitrary $\gamma \neq 0$, as shown in the right panel of Fig. S1(a). From Eq. S8, γ only decides the range of real-part-degenerate surfaces in k_z and does not change the surface shape. That means, the real-part-degenerate surfaces exactly illustrate the process of WPs evolving into WERs when increasing γ, as marked by the black arrows in the right panel of Fig. S1(a).

More specifically, Figs. S1(b)-S1(d) display the dispersions of the first two bands, on three representative planes colored green in the right panel of Fig. S1(a). The first

plane intersects with the imaginary-part-degenerate surfaces, and the bulk dispersion displays a ring-like degeneracy in the imaginary part, as shown in Fig. S1(b). The second plane intersects with WERs at the $k_z = k_{z0}$ plane, where the simultaneous degeneracy of the real and imaginary parts of bands occurs, as shown in Fig. S1(c). The third plane intersects with the real-part-degenerate surfaces, and the bulk dispersion exhibits a ring-like degeneracy in the real part, as shown in Fig. S1(d). The calculated bulk dispersions agree well with the theoretical prediction.

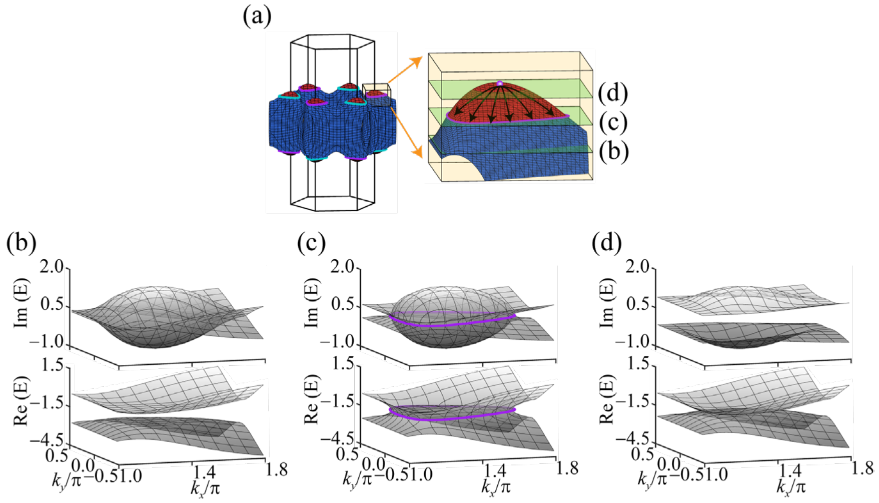

FIG. S1. Spectra degeneracies of the first two bands with loss applied to coupling t_0. (a) Left panel: spectral degenerate surfaces in the first BZ. Right panel: enlarged view of the yellow box in the left panel. The red and blue surfaces are the real- and imaginary-part degenerate surfaces, respectively. The purple and cyan rings are WERs connecting the real- and imaginary-part degenerate surfaces. The purple sphere in the right panel represents the WPs with $\gamma = 0$, and the black arrows show the evolution of one WP into one WER when increasing γ from 0 to 0.8. (b)-(d) Bulk dispersions of the first two bands on the green planes shown in the right panel of (a). The parameters are chosen as $t_0 = -1.385$, $t_1 = -1$, $t_2 = -0.6$ and $\gamma = 0.8$.

Second, we analyze the scenario where loss is applied to the coupling t_1. In this case, the distribution of spectral degeneracy can be derived following the procedure outlined as discussed above. For simplicity, we omit the redundant derivations and directly present the analytical results for spectral degeneracy with the selected

parameters. The real-part degeneracy is described by

$$\begin{cases} -\frac{\sqrt{t_0^2-\gamma^2}+t_1}{2t_2} \leq \cos k_z \leq \frac{t_0-t_1}{2t_2} \\ \cos k_x + 2\cos\frac{k_x}{2}\cos\frac{\sqrt{3}k_y}{2} = \frac{3t_0-9J_1}{4t_0} \end{cases}. \tag{S13}$$

The above equation describes the real-part degeneracy surface, whose distribution is illustrated by the red surface in Fig. S2(a). The imaginary-part degeneracy is described by

$$\begin{cases} \cos k_z \leq -\frac{\sqrt{t_0^2-\gamma^2}+t_1}{2t_2} \\ \cos k_x + 2\cos\frac{k_x}{2}\cos\frac{\sqrt{3}k_y}{2} = \frac{3t_0-9J_1}{4t_0} \end{cases}. \tag{S14}$$

The above equation describes the imaginary-part degeneracy surface, whose distribution is illustrated by the blue surface in Fig. S2(a). Notably, the boundary curves connecting the real- and imaginary-part degeneracy surfaces correspond to WERs (shown as purple and cyan rings), which are described by

$$\cos k_x + 2\cos\frac{k_x}{2}\cos\frac{\sqrt{3}k_y}{2} = C_0, \tag{S15}$$

where $C_0 = \frac{3t_0-9(t_1+2t_2\cos k_{z0})}{4t_0}$ with $\cos k_{z0} = -\frac{\sqrt{t_0^2-\gamma^2}+t_1}{2t_2}$. In other words, the WERs are located at $k_z = \pm\,\mathrm{acos}\frac{-\sqrt{t_0^2-\gamma^2}-t_1}{2t_2}$ planes. The right panel of Fig. S2(a) shows the spectral degeneracy distribution near the WER, where the black arrows illustrate the process of a WP evolving into a WER as γ increases. The results demonstrate that the evolution direction is opposite when loss is applied to coupling t_1 compared to the case of t_0.

More specifically, Figs. S2(b)-S2(d) display the dispersions of the first two bands on three representative planes colored green in the right panel of Fig. S2(a). In contrast to Fig. S1, Figs. S2(b)-S2(d) illustrate the opposite evolution of bulk dispersions: they first exhibit only real-part degeneracy, then evolve into a WER with degeneracy in both real and imaginary parts, and finally converge to purely imaginary-part degeneracy. This evolution is consistent with the theoretical predictions.

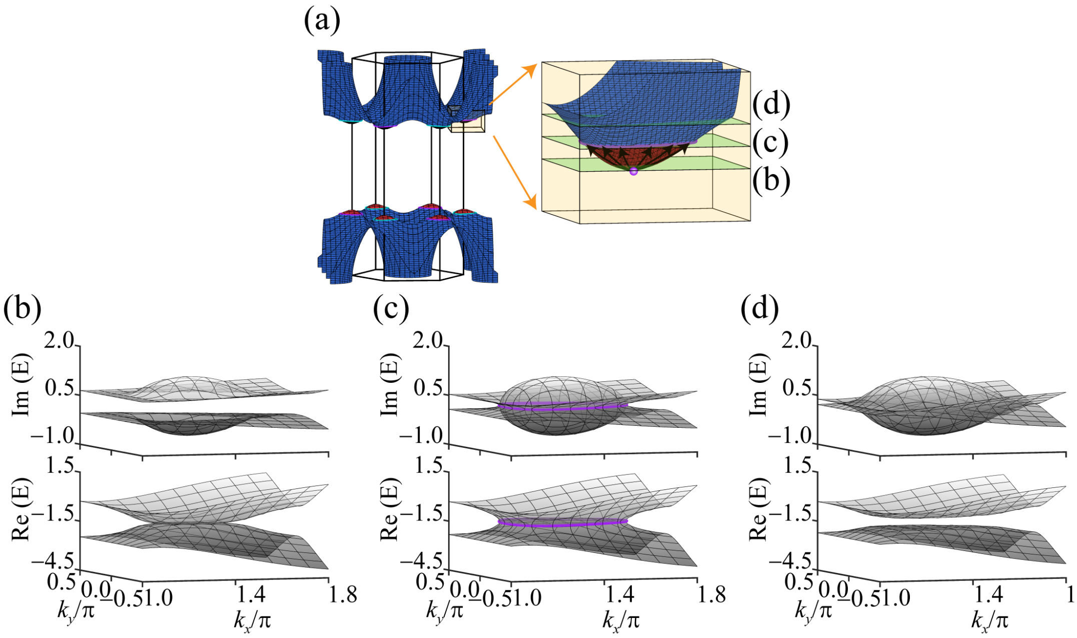

FIG. S2. Spectra degeneracies of the first two bands with loss applied to coupling t_1. (a) Left panel: spectral degenerate surfaces in the first BZ. Right panel: enlarged view of the yellow box in the left panel. The red and blue surfaces are the real- and imaginary-part degenerate surfaces, respectively. The purple and cyan rings are WERs connecting the real- and imaginary-part degenerate surfaces. The purple sphere in the right panel represents the WPs with $\gamma = 0$, and the black arrows show the evolution of one WP into one WER when increasing γ from 0 to 0.6. (b)-(d) Bulk dispersions of the first two bands on the green planes shown in the right panel of (a). The parameters are chosen as $t_0 = -1.385$, $t_1 = -1$, $t_2 = -0.6$ and $\gamma = 0.6$.

Third, when loss is applied to coupling t_2, the direction in which WPs evolve into WERs is the same as that when loss is applied to the coupling t_1. However, the resulting WERs is significantly smaller under the same loss strength. Following a similar derivation, the real-part degeneracy in this case is described by

$$\begin{cases} \frac{-t_1 t_2 + \sqrt{t_0^2 t_2^2 + t_0^2 \gamma^2 - t_1^2 \gamma^2}}{2(t_2^2 + \gamma^2)} \leq \cos k_z \leq \frac{t_0 - t_1}{2t_2} \\ \cos k_x + 2\cos\frac{k_x}{2}\cos\frac{\sqrt{3}k_y}{2} = \frac{3t_0 - 9J_1}{4t_0} \end{cases}. \tag{S16}$$

The above equation describes the real-part degeneracy surface, whose distribution is illustrated by the red surface in Fig. S3(a). The imaginary-part degeneracy is described

by

$$\begin{cases} \cos k_z \le \dfrac{-t_1t_2+\sqrt{t_0^2t_2^2+t_0^2\gamma^2-t_1^2\gamma^2}}{2(t_2^2+\gamma^2)} \\ \cos k_x + 2\cos\dfrac{k_x}{2}\cos\dfrac{\sqrt{3}k_y}{2} = \dfrac{3t_0-9J_1}{4t_0} \end{cases}. \tag{S17}$$

The above equation describes the imaginary-part degeneracy surface, whose distribution is illustrated by the blue surface in Fig. S3(a). Notably, the boundary curves connecting the real- and imaginary-part degeneracy surfaces correspond to WERs (shown as purple and cyan rings), which are described by

$$\cos k_x + 2\cos\frac{k_x}{2}\cos\frac{\sqrt{3}k_y}{2} = C_0, \tag{S18}$$

where $C_0 = \frac{3t_0-9(t_1+2t_2\cos k_{z0})}{4t_0}$ with $\cos k_{z0} = \frac{-t_1t_2+\sqrt{t_0^2t_2^2+t_0^2\gamma^2-t_1^2\gamma^2}}{2(t_2^2+\gamma^2)}$. In other words, the WERs are located at $k_z = \pm\,\mathrm{acos}\frac{-t_1t_2+\sqrt{t_0^2t_2^2+t_0^2\gamma^2-t_1^2\gamma^2}}{2(t_2^2+\gamma^2)}$ planes. The right panel of Fig. S3(a) shows the spectral degeneracy distribution near the WER. The results show that the evolution direction of the spectral degeneracy is the same as when loss is applied to coupling t_1. However, under the same loss strength, the WERs here is significantly smaller. This is because the magnitude of loss varies with k_z, and the effective loss near the WPs is $\frac{t_0-t_1}{t_2}\gamma$. Under the selected parameters, $\frac{t_0-t_1}{t_2} < 1$ so the WPs is less affected.

More specifically, Figs. S3(b)-S3(d) display the dispersions of the first two bands on three representative planes colored green in the right panel of Fig. S3(a). These figures exhibit the same bulk dispersion evolution as Fig. S2 (where loss is applied to t_1), but the resulting WERs are notably smaller. The calculated dispersions are in excellent agreement with the theoretical predictions.

Therefore, we conclude that WPs consistently evolve into WERs when loss is applied to different couplings, though the detailed evolution process varies with the specific couplings in question.

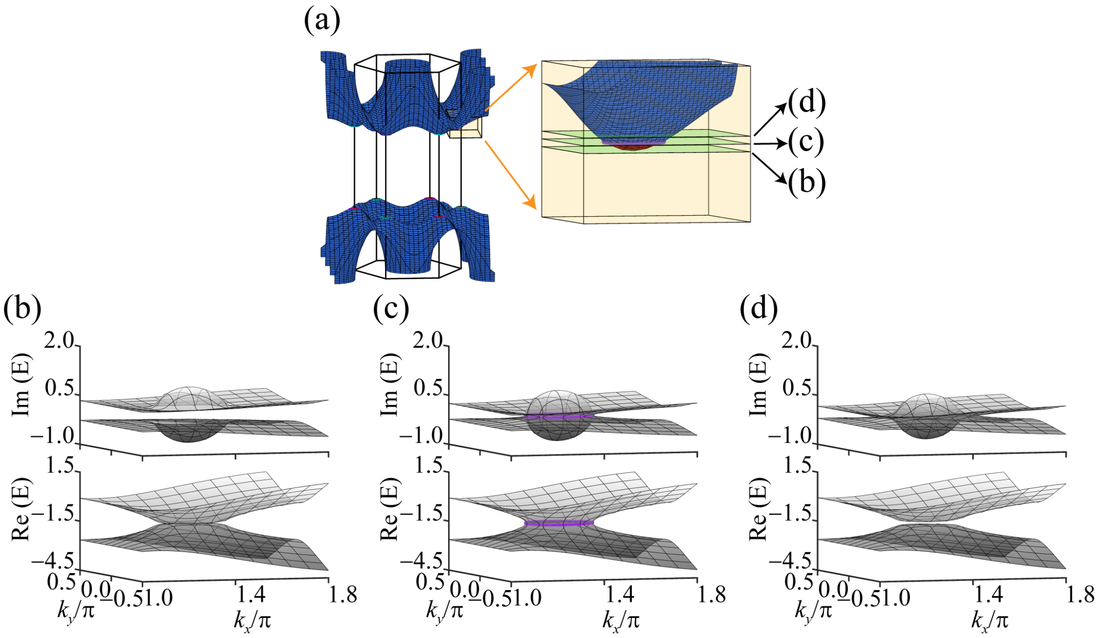

FIG. S3. Spectra degeneracies of the first two bands with loss applied to coupling t_2. (a) Left panel: spectral degenerate surfaces in the first BZ. Right panel: enlarged view of the yellow box in the left panel. The red and blue surfaces are the real- and imaginary-part degenerate surfaces, respectively. The purple and cyan rings are WERs connecting the real- and imaginary-part degenerate surfaces. (b)-(d) Bulk dispersions of the first two bands on the green planes shown in the right panel of (a). The parameters are chosen as $t_0 = -1.385$, $t_1 = -1$, $t_2 = -0.6$ and $\gamma = 0.6$.

S2. Fermi arc surface states

In this section, we calculate the Fermi arc surface states in the tight-binding model. We focus on the XZ surface of a ribbon geometry, as shown in Fig. S4(a). The ribbon contains 41 unit cells in the y direction and is periodic along the x and z directions. Figure S4(b) displays the surface dispersion, where the purple and cyan lines are projected WERs, and the green and gray surfaces denote the surface and bulk states, respectively. The surface states are gapless as a whole. Figure S4(c) displays the isofrequency contour of the surface states. The colormap is calculated by spectral function $A(E) = -\frac{i}{\pi}\mathrm{Im}G^r(E)$, where $G^r(E)$ is the retarded Green function and $E = -1.5$ is the real part of the energy of the WERs, respectively. The green lines clearly show that the projected WERs with opposite Chern numbers are connected by Fermi arc surface states.

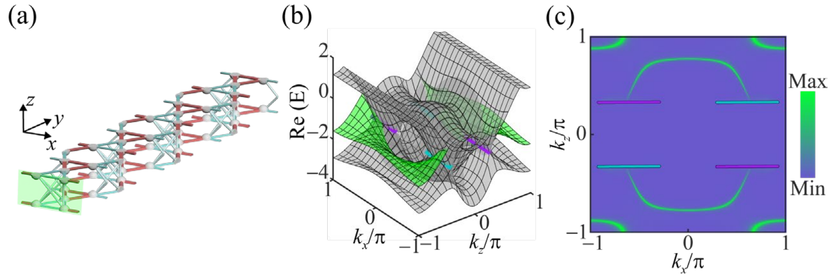

FIG. S4. Fermi arc surface states in the tight-binding model. (a) A ribbon geometry, which is periodic in the x and z directions. (b) Surface dispersion of the ribbon geometry. (c) Isofrequency contour at the WER energy, where the Fermi arc surface states connect the projected WERs with opposite Chern numbers.

S3. Spectral topology of ERs with different loss strengths

In this section, we calculate the spectral topology of exceptional rings (ERs) with different loss strengths γ. We first consider the case where $\gamma = 0.8$. In Fig. S5(a), we choose circle paths $\mathcal{L}_{1,2}$ as closed integral paths to calculate the spectral winding number v for two typical WERs in our tight-binding model. Path $\mathcal{L}_{1(2)}$ is centered on the WER at $k_z = \pm k_{\text{WER}}$, with a radius of 0.2π, the integration parameter is the angle θ in the anticlockwise direction, and the angle between the circular face surrounded by the path and the k_x axis is φ. The calculated spectra for the first two bands along paths $\mathcal{L}_{1,2}$ are displayed in Figs. S5(b) and S5(c), respectively. The eigenenergies wind anticlockwise (clockwise) for $\mathcal{L}_{1(2)}$ and form a closed loop in the complex plane, and the calculated winding number is $v = \pm 1$. As φ varies, the calculated $v = \pm 1$ are stable in Fig. S5(d), indicating that the winding numbers of the WERs are well quantized.

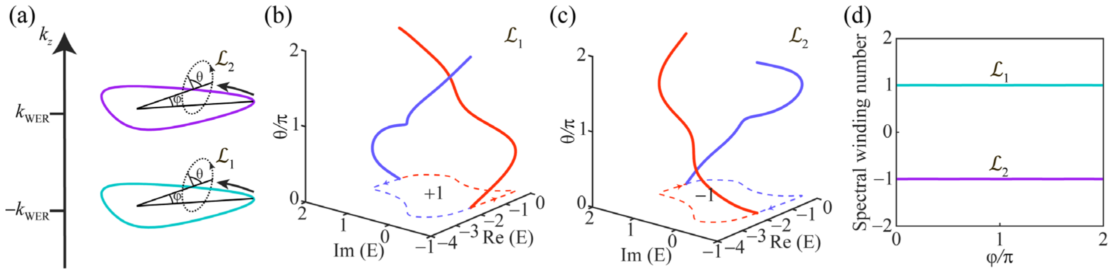

FIG. S5. Spectral winding number v of the WERs. (a) WERs and closed paths $\mathcal{L}_{1,2}$ encircling the typical WERs. (b) Spectral winding for the first two bands along the path $\mathcal{L}_1$ as a function of θ. $\varphi = 0°$ is set. (c) The same as (b) but for $\mathcal{L}_2$. (d) Calculated spectral winding numbers as φ varies.

We then consider the case with $\gamma = 1.5$. As shown in Fig. S6(a), when the loss strength is $\gamma = 1.5$ (a sufficiently large value), two WERs lying in the same plane in the BZ touch and merge into a single ER. The real and imaginary parts of the dispersion around the merged ERs are shown in Fig. S6(b). Using a method similar to that in Fig. S5(a), we calculated the spectral topology of the two merged ERs along paths $\mathcal{L}_3$ and $\mathcal{L}_4$ shown in Fig. S6(a). The results are presented in Fig. S6(c). Interestingly, the

spectral winding number of the merged ER in the $k_z = \pm k_{\mathrm{ER}}$ plane is opposite to that of the WER in the $k_z = \pm k_{\mathrm{WER}}$ plane.

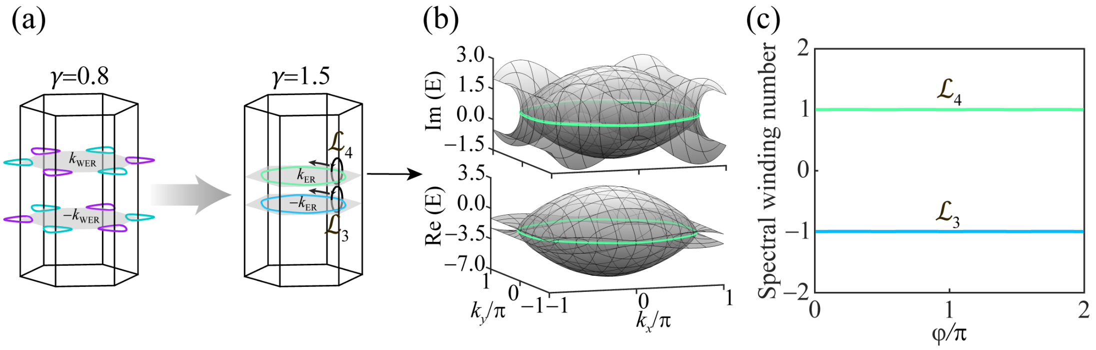

FIG. S6. Evolution of WERs with increasing loss. (a) Distributions of the WERs (left panel) with $\gamma = 0.8$ and merged ERs (right panel) with $\gamma = 1.5$ in the BZ. (b) Real and imaginary parts of the local dispersion around one merged ER in (a), as marked by the black arrow. (c) Calculated spectral winding numbers as φ varies.

S4. Non-Hermitian skin effects

The spectral topology in the WER semimetal prefigures the existence of non-Hermitian skin effects, which can be examined in detail by analyzing the spectral areas and the spectral winding along specific directions.

First, we discuss the skin effect for bulk states, which can be guaranteed by the non-zero spectral area of the complex energy spectrum of the unit cell, as shown in Fig. S7(a). Interestingly, the skin effect is geometry-dependent because the skin modes are forbidden along specific directions. Specifically, there exist mirror symmetries normal to the x (and equivalently, its threefold-rotation-related directions) and z directions. Owing to the mirror symmetries [e.g., $\mathcal{M}_x H(k_x, k_y, k_z)\mathcal{M}_x^{-1} = H(-k_x, k_y, k_z)$], the winding numbers along any paths parallel to the normal directions are zero [1], implying that the skin modes are forbidden along these directions. To be more explicit, we show the energy spectra along the routes $(k_x, 0, \pi/2)$ and $(\pi/4, 0, k_z)$ in Figs. S7(b) and S7(c), respectively, which are open arcs with zero winding numbers. In addition, although there exists no mirror symmetry along the y direction (and its equivalent directions), the skin modes are also forbidden due to the symmetry $SH(k_x, k_y, k_z)S^{-1} = H(k_x, -k_y, k_z)$, where $S = \begin{pmatrix} 0 & 0 & 1 \\ 0 & 1 & 0 \\ 1 & 0 & 0 \end{pmatrix}\mathcal{K}^t$ and $\mathcal{K}^t$ is the transposition operation. On the contrary, spectra along other directions can exhibit point gaps, as exemplified in Fig. S7(d), which implies the existence of skin modes along the $(k_x, \pm\pi/\sqrt{3}, k_x - \pi/3)$ direction.

Then we discuss the skin effects for topological surface states, i.e., hinge-dependent second-order skin effects in our system. Not to lose the generality, we focus on the XZ surface states. Figure S7(e) displays the spectrum of a ribbon geometry, which is periodic along the x and z directions. The surface states are colored green, with a non-zero spectral area. In Figs. S7(f)-S7(h), we show the energy spectra along specific routes ($k_z = \pi/2$, $k_x = \pi/4$, and $k_z = k_x - \pi/3$) in the surface BZ. Similar to the aforementioned scenario, point gaps are selectively forbidden along the k_x and k_z directions, implying that the second-order effect is hinge-dependent.

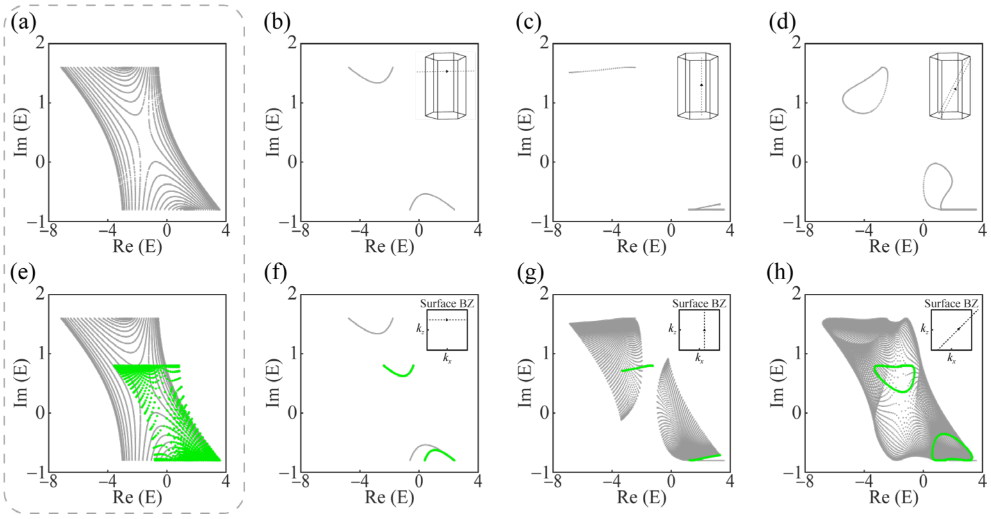

FIG. S7. Complex energy spectra for the bulk and surface states. (a) Complex energy spectrum of the bulk states, obtained from the unit cell of the tight-binding model. (b)-(d) Complex energy spectra along specific routes in the BZ, as depicted in the insets. (e)-(h) The same as (a)-(d) but for the XZ surface states, obtained from a ribbon geometry which is periodic in the x and z directions. The gray and green dots denote bulk and surface states, respectively.

To verify the existence of skin effects, below we calculate the spectra of two different geometries under fully open boundary conditions. Figure S8(a) displays the spectrum of the rhombic prism geometry in Fig. 1(a) of the main text, which contains $15 \times 15 \times 13$ unit cells. The spectral areas are the same as those in Fig. S7, implying the absence of skin modes. Figure S8(b) shows the distribution of all eigenstates defined as $W_b(j) = \frac{1}{N_b}\sum_{n_b}\left|\psi_{n_b}(j)\right|^2$, where $\psi_{n_b}(j)$ is the n_b-th eigenstate at site j, and N_b denotes the total number of eigenstates. The colormap shows that the distribution of eigenstates remains extended and no skin effect occurs. We also select the XZ (and its equivalent) surface states and display their distribution in Fig. S8(c), which is defined as $W_s(j) = \frac{1}{N_s}\sum_{n_s}\left|\psi_{n_s}(j)\right|^2$ with $\psi_{n_s}(j)$ being the n_s-th surface state at site j and N_s denoting the total number of surface states. The colormap shows an extended distribution on the focused surfaces without hinge-localized skin modes. These results show that the skin effects are forbidden in this geometry for both bulk and surface states.

Then we calculate the spectrum of a tilted prism geometry with $[\bar{2}112]$-directional hinges as discussed in Fig. 1(h) of the main text, which contains $13 \times 17 \times 13$ unit cells. One can see from Fig. S8(d) that, now the spectral areas undergo a deformation compared to Fig. S7, which prefigures the existence of skin modes for both bulk and surface states. Figure S8(e) shows the distribution of W_b, which exhibits a significant localization on the left and right surfaces normal to $[2\bar{1}\bar{1}2]$ direction as expected. Figure S8(f) shows the distribution of W_s for XZ surface states, which selectively localize at $[\bar{2}112]$-directional hinges.

These results confirm the presence of surface-dependent skin effect and hinge-dependent skin effects in our system, as the first-order skin effect and second-order skin effect, respectively.

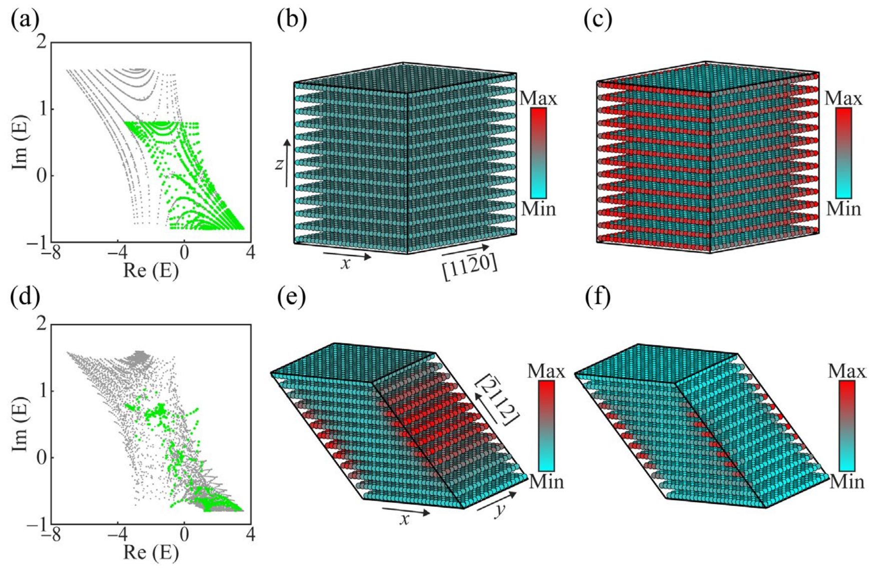

FIG. S8. Surface-dependent (first-order) skin effect and hinge-dependent (second-order) skin effect. (a) Complex energy spectrum of the rhombic prism geometry. The XZ (and its equivalent) surface states are highlighted in green. (b) Distribution of all eigenstates W_b. (c) Distribution of all surface states W_s. (d)-(f) The same as (a)-(c) but for a tilted prism geometry with $[\bar{2}112]$-directional hinges.

Finally, we show the skin effect becomes increasingly localized as the loss strength increases. Figures S9(a)-S9(c) show the eigenstate distributions for all states in the tilted prism geometry with loss strengths $\gamma = 0.2$, 0.8, and 1.5, respectively. In all three

cases, the bulk states exhibit the skin effect localized to the left and right surfaces. One can see that the skin effect is weak at $\gamma = 0.2$, becomes pronounced at $\gamma = 0.8$, and is significantly enhanced at $\gamma = 1.5$, demonstrating that the skin effect of bulk states is strengthened as the non-Hermitian strength increases. Meanwhile, Figures S9(d)-S9(f) display the eigenstate distributions of the XZ surface states for $\gamma = 0.2,\ 0.8$, and 1.5. It can be observed that the surface states also become more localized on the side hinges with increasing non-Hermitian strength, showing an enhanced skin effect as well.

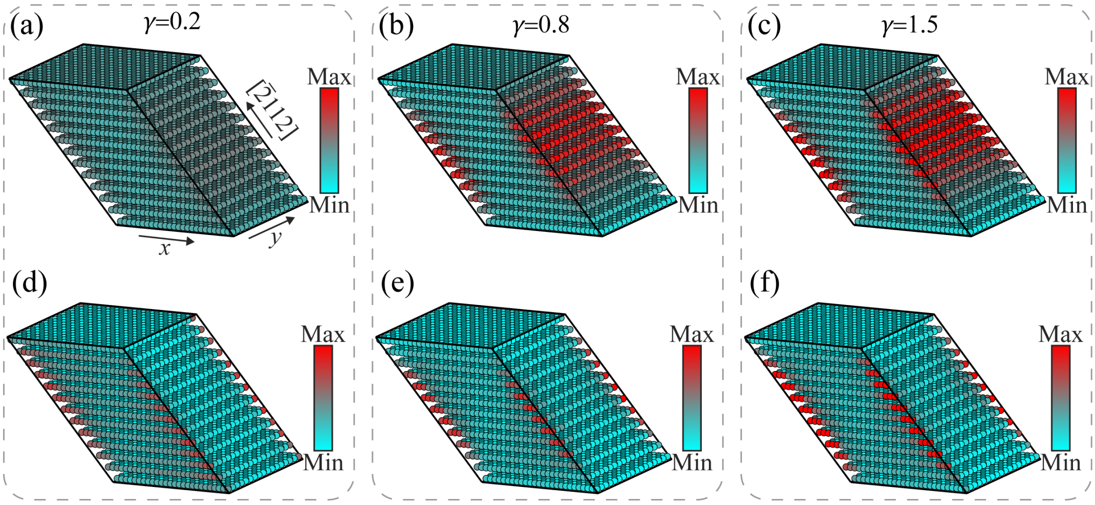

FIG. S9. Strengthened skin effect with increasing loss strength γ. (a)-(c) Distributions of all eigenstates in the tilted prism geometry with $\gamma = 0.2,\ 0.8$, and 1.5, respectively. (d)-(f) Distributions of all XZ surface states in the tilted prism geometry with $\gamma = 0.2$, 0.8, and 1.5, respectively.

S5. Bulk polarization with different loss strengths

In this section, we discuss that the bulk polarization remains unaffected by increasing loss and its phase transition points in k_z always coincide with the positions of the WPs/WERs/ERs. Figures S10(a) and S10(b) depict the evolution of the bulk polarization with k_z and the hinge dispersion with $\gamma = 0$, respectively. It can be observed that the phase transition points of bulk polarization are $k_z = \pm k_{\mathrm{WP}}$, and topological hinge states exist within the region of non-zero bulk polarization. Figures S10(c)-S10(d) and S10(e)-S10(f) present the corresponding results with $\gamma = 0.8$ and $\gamma = 1.5$, respectively. Notably, the bulk polarization takes $k_z = \pm k_{\mathrm{WER}}/k_{\mathrm{ER}}$ as its phase transition points, and the topological hinge states persist in the topologically non-trivial region. Interestingly, the imaginary part of the energy of the topological hinge states remains zero with increasing loss, demonstrating that these states are completely immune to loss.

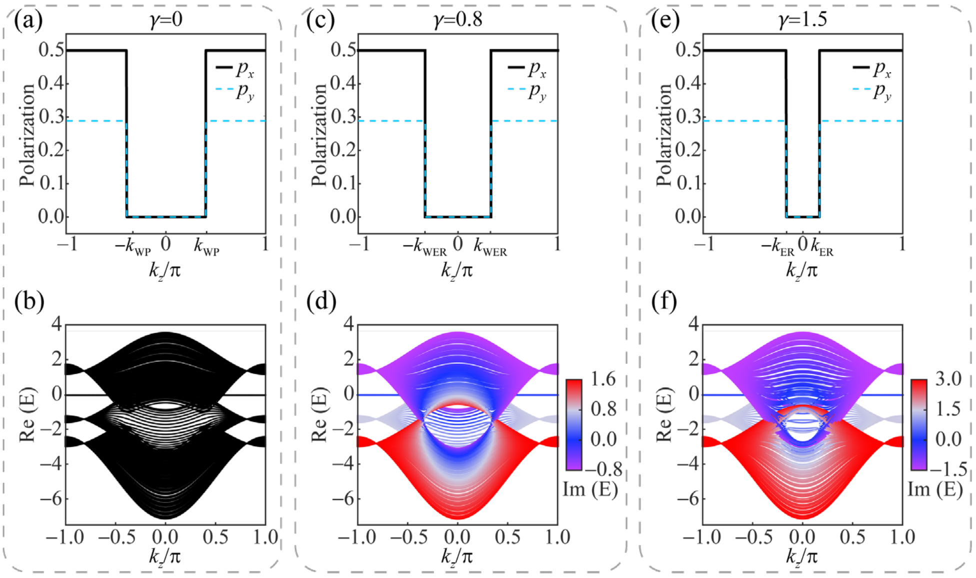

FIG. S10. Bulk polarization unaffected by increasing loss strength. (a) Bulk polarization (p_x, p_y) of the lowest band as a function of k_z with $\gamma = 0$. (b) Hinge dispersion along the k_z direction with $\gamma = 0$. (c) and (d) The same as (a) and (b) but for the case with $\gamma = 0.8$. (e) and (f) The same as (a) and (b) but for the case with $\gamma = 1.5$. The colormap in (d) and (f) represent the imaginary part of hinge dispersion.

S6. Designed loss in AM sample

In this section, we show how the loss is added in the AM sample. In simulations, we constructed two rectangular surfaces on the wall of each intercell connecting tube, and we applied impedance boundary conditions on them. When the acoustic wave is transmitting in the sample, part of which would transmit into free space by the impedance boundary surfaces, so the two surfaces can play the role to provide loss to the AM sample in the simulation [2]. In experiments, we constructed two rectangular holes with the same size in the wall of each intercell coupling tube, and filled sponges into the holes. When the acoustic waves propagate in the sample, part of which would be absorbed, so the two holes with filled sponges can provide loss in experiments.

To quantify the loss intensity, we fabricate a structure of two coupled cavities, as shown in Fig. S11(a), which has the same cavities and connecting tubes as those in the AM sample. To evaluate the global loss, we first measure the response spectrum for the coupled cavities without sponge-filled holes, with respect to the source (detector) placed at the top (bottom) of the left (right) cavity. The measured result is given by the blue curve in Fig. S11(b), and the black curve is the fitting curve obtained in simulations with a global loss of $3i$ m/s applied to the sound speed (with the real part 340 m/s). Similarly, to evaluate the designed loss, we measure and fit the response spectrum for the coupled cavities with sponge-filled holes, as shown in Fig. S11(c). The size of each hole is set as $s = 4.4 \times 3.8\ \mathrm{mm}^2$, which is imposed by impedance boundary conditions in simulations with acoustic impedance $1500\ \mathrm{Pa \cdot s/m}$.

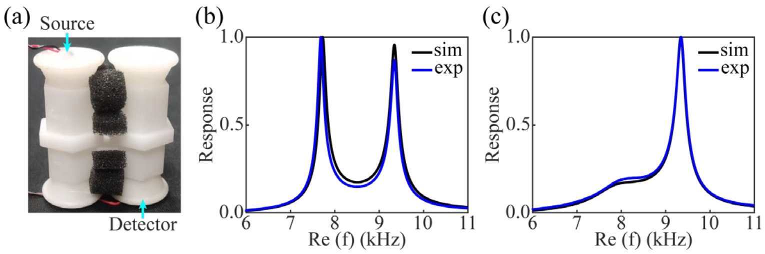

FIG. S11. Designed loss in AM sample. (a) A structure of two coupled cavities used to quantify the global loss and the designed loss in the waveguide. The connecting tube are filled sponges (black). (b) and (c) Response spectra of the coupled cavities without and with sponge-filled holes, respectively. The black and blue curves represent simulated and measured results, respectively.

S7. Spectral winding number of the acoustic WERs

In this section, we show the spectral winding of the acoustic WERs. Figure S12(a) displays the first BZ of the AM, where the purple and cyan rings are acoustic WERs with different Chern numbers. There are four closed paths $\mathcal{L}_{1-4}$ (black circles) enclosing the four WERs counterclockwise, as shown in Fig. S12(a). The complex spectra along the four paths are shown in Figs. S12(b)-S12(e), where the first two bands along the paths $\mathcal{L}_{1,2}$ wind clockwise in the complex plane, corresponding to the winding number -1. Bands along the paths $\mathcal{L}_{3,4}$ wind counterclockwise in the complex plane, corresponding to the winding number 1. Therefore, the acoustic WERs in the $k_z = \pm k_{\text{WER}}$ planes have the spectral winding number ∓ 1, which is consistent with the tight-binding model.

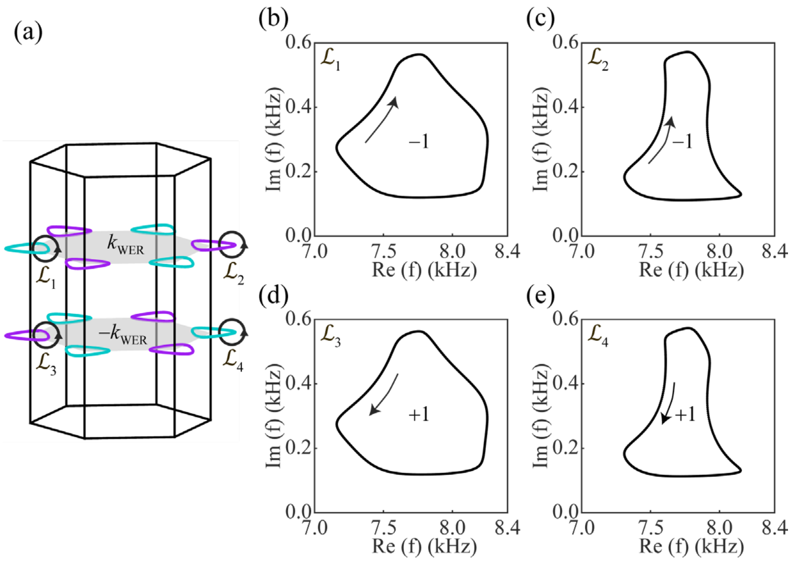

FIG. S12. Spectral winding number of WERs in AM. (a) Distribution of the acoustic WERs in the first BZ. Purple (cyan) rings represent WERs with Chern number 1 (-1). The shaded planes represent the $k_z = \pm k_{\text{WER}}$ planes. The circular paths $\mathcal{L}_{1-4}$ are selected to calculate the spectral winding number. (b)-(e) Simulated spectra of the first two bands along the paths in (a).

S8. Non-Hermitian skin effects for acoustic waves

In this section, we discuss the skin effects of bulk and surface states in AM. For the bulk states, the skin effects can be guaranteed by the non-zero spectral area of the unit cell, as shown in Fig. S13(a). For the XZ surface states, the skin-topological effects can be guaranteed by the non-zero spectral area of the ribbon structure (periodic along the x and z directions), as shown in Fig. S13(b). Figure S13(c) displays the spectrum of the rhombic prism AM, which contains $8 \times 8 \times 8$ unit cells. Similar to the tight-binding results, the spectral areas of the bulk and surface states imply the absence of skin modes. Figure S13(d) shows the distribution of all eigenstates, which remains extended and no skin effect occurs. We also select the XZ (and its equivalent) surface states and display their distribution in Fig. S13(e), which shows extended distribution on the focused surfaces without hinge-localized skin modes. The results show no skin effects in this AM for both bulk and surface states.

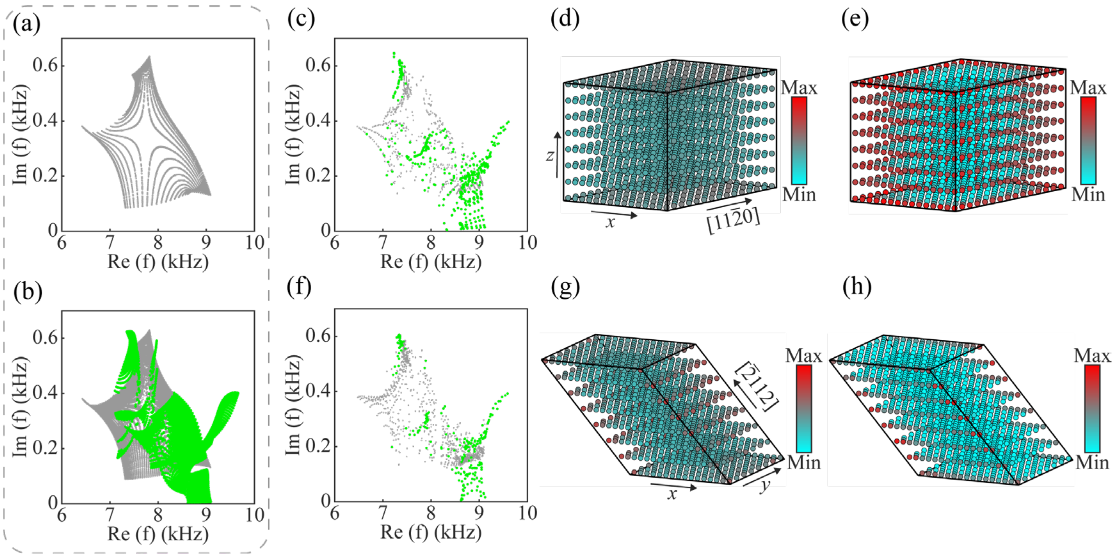

FIG. S13. Surface-dependent (first-order) skin effects and hinge-dependent (second-order) skin effects in AMs. (a) and (b), Complex energy spectrum of the unit cell and ribbon, respectively. The gray and green dots denote bulk and XZ surface states, respectively. (c) Complex energy spectrum of the rhombic prism AM. (d) Distribution of all eigenstates of the rhombic prism AM. (e) Distribution of surface states of the rhombic prism AM. (f)-(h) The same as (c)-(e) but for a tilted prism AM.

Then, we calculate the spectrum of a tilted prism AM, which also contains $8 \times 8 \times 8$ unit cells. As we can see from Fig. S13(f), the spectral areas undergo deformations compared to those in Figs. S13(a) and S13(b), which prefigure the existence of skin effects for both bulk and surface states. Figure S13(g) shows the distribution of all eigenstates, which exhibits obvious localization on the left and right surfaces normal to the $[2\bar{1}\bar{1}2]$ direction. Figure S13(h) shows the distribution of surface states in the XZ surfaces, which are selectively localized at $[\bar{2}112]$-direction hinges. These results indicate the presence of geometry-dependent skin effects in AM, which are consistent with the tight-binding results.

S9. Robustness of the topological hinge states against perturbations

Since the topological hinge states are protected by a nonzero bulk polarization, they remain robust against random disorder. Below, we experimentally demonstrate this point by investigating the effect of disorder, which is introduced by adding structural disorder to the acoustic cavities at the left hinge of the acoustic metamaterial (AM). As shown in the left panel of Fig. S14(a), four cylindrical structures are installed on the wall of each hinge cavity, with a diameter of 3.4 mm and a height randomly distributed between 1-7 mm. Due to the wall thickness of 4 mm, cylinders of different sizes partially reduce or increase the volume of the air cavity, thereby altering the resonant frequencies. As such, disordered hinge on-site potentials are effectively introduced to the hinge cavities. We examine four sets of randomly chosen disorder structure (disorder 1-4) and measure the hinge response spectra and the hinge dispersions, as shown in Figs. S14(b)-S14(f). One can see that the measured spectra of the disordered AM show negligible deterioration compared to those without disorder, indicating the robustness of the hinge states to small perturbations.

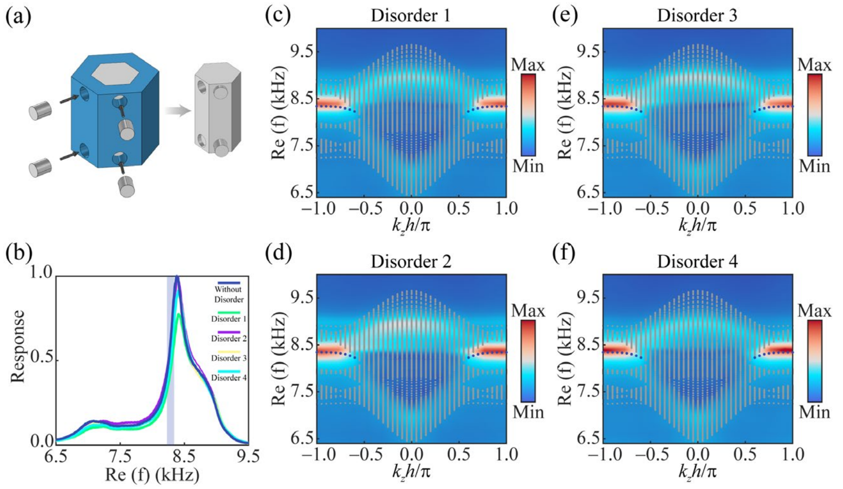

FIG. S14. Robustness of the topological hinge states against perturbations. (a) Experimental setup. Left panel: The gray hexagonal prism represents the air cavity, and the blue outer shell is a wall with a thickness of 4 mm. Disorder is introduced by randomly inserting cylinders of different lengths into the four holes on the wall. Right panel: The resulting air cavity after inserting cylinders of different lengths. (b) Measured response spectra for the left hinge of the AM without or with disorders. (c)-(f) Measured hinge dispersion (colormap) for the left hinge with four sets of disorders.

S10. Observation of the trivial hinge states

In this section, we show the observation of the trivial hinge states in the AM sample. As discussed in the main text and illustrated in Fig. S15(a), there exist two branches of trivial hinge states at around 7.24 and 8.90 kHz. Different from topological hinge states, these trivial hinge states localize at the right hinge of the sample and have larger imaginary parts of frequencies which can lead to weaker signals of acoustic waves propagating along the hinge. Therefore, we place the source at the center of the right hinge and measure the hinge dispersion, as shown in Fig. S15(b). Influenced by the system loss, the experimental data is quite noisy, especially for the first branch of hinge states around 7.24 kHz with relatively larger imaginary parts. We also display the measured hinge fields at 7.24 and 8.90 kHz in Figs. S15(c) and S15(d), respectively. One can see that the hinge propagation for acoustic waves is weaker than that in Fig. 4(a) in the main text, reflecting the difference between the trivial hinge states and topological ones.

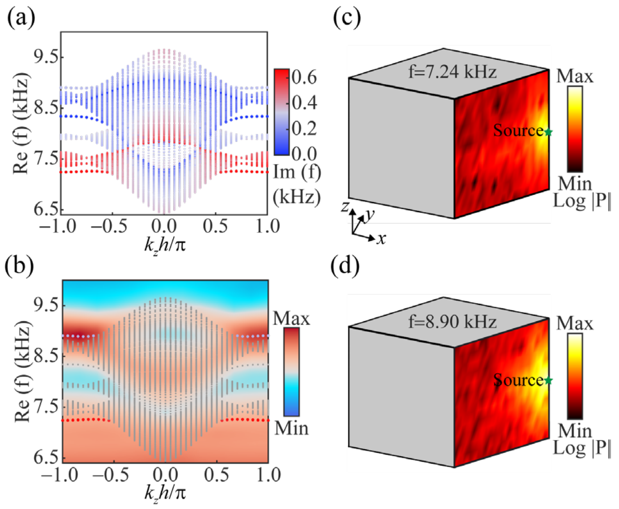

FIG. S15. Observation of the trivial hinge states in the AM sample. (a) Simulated hinge dispersion of the sample, which is the same as Fig. 4(a) in the main text. The THSs around 8.34 kHz are localized at the left hinge, and the trivial hinge states around 7.24 kHz and 8.90 kHz localize at right hinges. (b) Measured hinge dispersion (colormap) for the right hinge. (c) and (d), Measured hinge field distributions (colormaps) at 7.24 kHz and 8.90 kHz, respectively. The source (green star) is placed at the center of the right hinge.

S11. Methods

Theoretical analysis

In Fig. 1(f) of the main text, we calculate the hinge dispersion using a rhombic prism geometry, which is periodic in the z direction and has 13×13 unit cells in the x-y plane. In Fig. 1(g), we use a ribbon geometry which is periodic in the x and z directions but finite in the y direction with 61 unit cells. In Fig. 1(h), the fully open boundary geometry has $13 \times 17 \times 13$ unit cells in the x, y, and $[\bar{2}112]$ directions. We distinguish the surface and bulk states based on the distribution features of their wavefunctions.

Numerical simulation

All the simulations are performed by the commercial COMSOL Multiphysics solver package, where the sound speed and air density are $340\ \mathrm{m/s}$ and $1.29\ \mathrm{kg/m^3}$, respectively. We choose the dipole mode along the z direction of the acoustic cavity to map the site in tight-binding model, and all the couplings are negative. In Figs. 3(c) and 3(d), we calculate the surface dispersion of the AM using a ribbon geometry with 21 unit cells in the y direction. In Figs. 4(c) and 4(d), we calculate the hinge dispersion of the AM using a rhombic prism geometry containing 13×13 unit cells in the x-y plane.

Experimental measurement

The experimental AM sample in this work is fabricated by 3D printing technology. The wall thicknesses of the cavities and waveguides without holes are $2\ \mathrm{mm}$, and those of waveguides with holes are $4\mathrm{mm}$. To fill sponges into the holes, the AM sample is fabricated layer by layer and then stacked via additional splicing structures. The acoustic field distributions shown in Figs. 2-4 and S14 are extracted from the top of each cavity, where the sources are placed in the center cavities of the sample's bulk (surface, hinges). The measured dispersions are obtained by Fourier transforming the corresponding acoustic fields.

REFERENCES

1 Zhang K, Yang Z and Fang C. Universal non-Hermitian skin effect in two and higher dimensions. *Nat Commun* 2022; **13**: 2496.

2 Yang Y, Jia H and Bi Y *et al*. Experimental demonstration of an acoustic asymmetric diffraction grating based on passive parity-time-symmetric medium. *Phys Rev Appl* 2019; **12**: 034040.
